# Immune Activation Following Spinal Cord Injury: A Review Focused on Inflammatory Changes in the Spinal Cord

**DOI:** 10.3390/ijms26199624

**Published:** 2025-10-02

**Authors:** Diogo Nascimento, Ana Ferreira, Célia Duarte Cruz

**Affiliations:** 1 Department of Biomedicine, Experimental Biology Unit, Faculty of Medicine of Porto, University of Porto, Alameda Prof. Hernâni Monteiro, 4200-319 Porto, Portugal; desn@sapo.pt (D.N.); anapf31@gmail.com (A.F.); 2 Department of Otolaryngology, Unidade Local de Saúde de Entre o Douro e Vouga, 4520-211 Santa Maria da Feira, Portugal; 3 Pain Neurobiology, Instituto de Investigação e Inovação em Saúde—i3S and IBMC, University of Porto, 4200-135 Porto, Portugal

**Keywords:** spinal cord injury, inflammation, immune cells

## Abstract

Spinal cord injury (SCI) poses a substantial physical, psychological and social burden. Although many therapies are currently available, it is still impossible to fully restore the lost organic functions of SCI patients. An important event in SCI physiopathology is the development of a neuron-repulsive fibrotic scar at the lesion site, a barrier that hampers neuronal growth and contributes to long-term functional impairment. This neuron-repulsive scar is present in severe spinal cord injuries in humans but is absent in some animals capable of natural regeneration. In humans and other mammals, various immune cells take part in the development and maturation of the glial scar, and cytokines and other molecular factors regulate the associated histologic changes. Pro-inflammatory cytokines and complement system proteins tend to be overexpressed early after SCI, but anti-inflammatory cytokines also participate in the remodelling of the injured tissue by regulating the excessively pro-inflammatory environment. This inflammatory regulation is not entirely successful in humans, and inflammation inhibitor drugs offer promising avenues for SCI treatment. Some non-specific immunosuppressor drugs have already been studied, but targeted modulation therapies may be more efficient and less prone to secondary effects. Continued experimental research and clinical trials are vital to advance findings and develop effective treatments, aiming to overcome the barriers to spinal cord regeneration and improve recovery for SCI patients.

## 1. Introduction

Spinal cord injury (SCI) is a damaging event that frequently leads to a debilitating physical status, causing motor, sensory, autonomic, and immune dysfunctions. With a global age-standardized incidence rate of 12 per 100,000 in 2019, SCI has presented an increasing trend over the last 3 decades among developing countries [1]. SCI is an undeniable physiological, psychological and financial problem affecting individuals, as well as society, and the massive disease burden associated with SCI has prompted a stunning crescendo of research works over the last couple of decades.

SCI is a broad designation that includes spinal damage of traumatic and non-traumatic origin [2]. Non-traumatic causes of SCI can be further divided into congenital, genetic or acquired [3] and include several clinical entities with great epidemiologic relevance, such as multiple sclerosis, vertebral neoplasms and spinal dysraphism [2,3]. Currently, treatments for SCI are based on three main pillars: drug therapies, stem cell-based therapies and surgical approaches [4]. These strategies have extensively improved the quality of life of SCI patients by improving symptoms and preventing damage progression. However, no therapy has hitherto been able to fully repair the injured spinal cord and completely restore motor, sensory, autonomic, and immune functions in a human patient.

The importance of better understanding the underlying pathophysiologic mechanisms of SCI is unquestionable if we aspire to develop new effective therapies. A central piece of SCI pathophysiology lies in the generation of a glial scar and its interaction with inflammatory cells and mediators, which play a critical role in morphophysiological changes in the structure of the spinal cord after SCI. The role of immune cell infiltration in the spinal cord injury tissue following SCI is extensively reported in the literature. These cellular populations, which include, inter alia, granulocytes, lymphocytes and macrophages, play a starring role in the formation of the fibrotic scar at the lesion site [5]. In addition to the well-known importance of this immune cell infiltration, in recent years, scientists have brought to light new immune mechanisms that seem to vastly influence spinal cord remodelling following SCI. Among these mechanisms, there is particularly convincing evidence demonstrating the importance of cytokine cascades, the complement system and macrophage phenotypes.

The present review will mainly focus on traumatic SCI, due to greater homogeneity and reproducibility in animal research, as well as a wide availability of original works. It aims to provide a summarized perspective of the recent findings concerning the critical role of the immune system cells and molecular factors in the spinal cord tissue remodelling following SCI. We also briefly refer to new and exciting animal models of SCI that offer hope for developing new treatments.

## 2. Materials and Methods

To produce this narrative review, available articles were searched in PubMed using the terms “spinal cord injury”, “inflammation”, “immune cells”, “glial scar” and “experimental animals”. No time limits were imposed.

## 3. Results

### 3.1. The Spinal Cord Lesion and the Development of the Scar

Immediately after the physical impact affecting the spinal cord, several changes can be found at macro- and microscopic levels—including the primary injury, caused by mechanical tissue disruption [5,6]. Damage of blood vessels is paramount as it leads to disruption of the blood–spinal cord barrier, ischemia and edema, and is accompanied by physical disruption of tissue architecture and direct impairment of function of neurons and glial cells [7,8]. Direct neuronal damage is often accompanied by an anterograde degeneration process, named Wallerian degeneration [9,10,11]. Impairment of microvasculature, neuronal degeneration and associated glial damage precipitate the generation of a pro-inflammatory and neuron-repulsive molecular environment [12]. This peculiar molecular environment is responsible for extensive tissue remodelling that leads to the formation of a fibrotic scar (during secondary injury) that blocks the connection between neuronal circuits rostral and caudal to the lesion. Regrowth of neuronal processes and full restoration of spinal cord architecture is impossible in most mammals due to the neuron-repulsive features of the glial scar. However, simply ablating chronic glial scars does not induce neuronal regrowth [13]. In fact, the astrocytic scar presents as a good medium for axonal growth when enriched with growth factors delivered via hydrogel, early after SCI [13]. Taking this into account, we need deep knowledge regarding the cellular and molecular components of the glial scar to manipulate its function. The development of this fibrotic scar depends on the recruitment of several cellular types that integrate the lesion site at different timings. These cells include astrocytes, fibroblasts, microglia, neural stem cells, pericytes, macrophages, neutrophils, and lymphocytes.

The secondary injury is initiated by molecular mediators that induce a local state of reactive astrogliosis and microglial activation. Cytokines produced by these resident cells further recruit and activate other astrocytes and microglia, but also recruit peripheral immune cells, as the blood–spinal cord barrier is disrupted [14,15]. The first peripheral immune cells to arrive at the lesion site are thought to be neutrophils (3–6 h after SCI), which peak during the first day post-injury [14,15,16]. Lymphocytes, fibroblasts and peripherally derived macrophages take longer to infiltrate the spinal cord but persist at the lesion site for weeks or even months in the spinal cord parenchyma [15,17].

**Figure 1:**
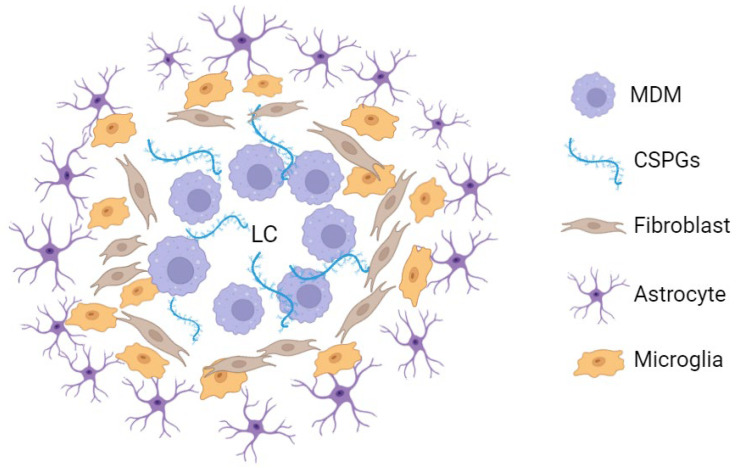
Simplified representation of the mouse spinal cord chronic glial scar cellular and extracellular components [5,17]. Around the lesion centre, macrophages proliferate in a chondroitin sulphate proteoglycan-enriched environment. Externally, microglial cells are found surrounded by an outer astrocyte layer. In rats and humans, the fibroblast layer represented in this figure is not always present (or at least not to this extent) [5,15,17]. LC: lesion centre; MDM: monocyte-derived macrophage; CSPGs: chondroitin sulphate proteoglycans. This figure was created using BioRender.com (accessed on 9 June 2024).

It is widely accepted that cellular populations involved in the generation of the glial scar are not stochastically distributed but instead respect a specific organization (Figure 1). In the mature scar, observed at chronic stages of the disease, a collagenous extracellular matrix is found surrounding the lesion core and incorporating peripherally derived macrophages. External to this layer, microglial cells are present, surrounded by an additional external rim of astrocytes, arranging their cellular processes around the lesion boundary (Figure 1) [5,6,15,18,19,20]. In mice, fibroblasts are also present. One to two weeks after spinal cord injury, the lesion site is sealed, and the glial scar is in active maturation [6]. This scar is currently viewed as an ever-changing dynamic structure that plays a central role in the spinal cord injury functional sequelae [21].

### 3.2. Immune Cellular Populations Permeating CNS

During injury and development of scar tissue, there is massive activation of the immune system by activation of resident glial cells and migration of peripheral immune cells. Several cellular populations are involved in the setting of secondary spinal injury. Resident cells, such as microglia and astrocytes, proliferate and change the molecular environment by producing pro-inflammatory cytokines [14,15]. This new extracellular environment, enriched in inflammatory mediators, induces the arrival of peripherally derived immune cells to the injury site, which has been facilitated by the mechanical disruption of blood vessels upon mechanical trauma of the spinal cord [14,15]. The most relevant immune cellular populations are described below (Table 1).

#### 3.2.1. Neutrophils

Neutrophils represent the most abundant type of leukocytes populating the peripheral blood. These cells have a multilobulated nucleus and possess enzyme-rich cytoplasmic granules, being classified as granulocytes, along with eosinophils and basophils [27,28]. Neutrophils are the first peripherally derived immune cells to invade the spinal injury site, following SCI, taking no longer than a few hours to permeate CNS and peaking around 24 h post-injury [28]. This was demonstrated by an increase in myeloperoxidase and defensin levels in the spinal cord tissue of rats and humans [14,15,16]. This early arrival of neutrophils is not unexpected, since these cells are regarded as the major cellular arm of the innate immune system [29]. By constantly patrolling the tissues for signs of invading pathogens, neutrophils can swiftly remove microorganisms and produce pro-inflammatory cytokines [28,30]. This attempt to sterilize the lesion site, preparing the spinal cord for repair, is, however, overtaken by the deleterious infiltration of an excessive neutrophilic population [16].

Neutrophil-induced neuroinflammation and edema in SCI have recently been linked to neutrophil extracellular traps (NETs) [31]. NETs are grid-like structures composed of nucleic acids and proteins that undergo externalization from the surface of neutrophils [32]. The formation of these traps constitutes one of the main antimicrobial actions of neutrophils, along with degranulation and phagocytosis [27]. NET formation mechanisms have recently drawn the attention of the scientific community and seem to pose a great therapeutic target in CNS pathology [33]. These mechanisms play an important role in the pathophysiology of stroke and CNS trauma and their inhibition improves neurologic outcomes in animal models of CNS injury [33].

While the presence of neutrophils in neuronal tissue after SCI is well established, studies exploring leukocyte counts in cerebrospinal fluid (CSF) are scarce. However, in the 1990s, a retrospective analysis showed an increase in CSF white blood cell count (WBCc) in SCI human patients, peaking 3.5 days after injury [23]. The WBCc gradually decreased after that time point. Polymorphonuclear cells, which include neutrophils, were the most prevalent cellular component, with a mean differential WBCc of 57.8%, potentially reflecting an inflammatory response to injury and associated blood–spinal cord barrier damage. This is likely a feature maintained in all mammals, as a study conducted in horses showed that equines with spinal cord compression and/or axonal degeneration tend to present neutrophils in the CSF more often than clinically normal horses [34]. A recent systematic review showed a non-statistically significant tendency for a higher blood concentration of neutrophil markers in SCI patients, in comparison to healthy individuals [22]. Accordingly, neutrophil chemoattractants, such as CXCL1, CXCL5 and CXCL6, are elevated in the blood serum of SCI patients [35,36]. Eosinophils and basophils seem to play a less important role than neutrophils in the setting and functional recovery of SCI [5,15,22,37], further reinforcing the involvement of neutrophils in SCI-related neuroinflammation.

#### 3.2.2. Lymphocytes

Adaptive immunity deeply relies on lymphocytes and is critical during spinal cord repair [38]. Classically, lymphocyte populations have been divided into three categories: T-cells, B-cells and natural killer (NK) cells [39]. T-cells have been extensively studied in the context of SCI, but all lymphocyte categories seem to play a role in SCI. T lymphocytes take longer than neutrophils to invade the spinal damaged tissue, peaking 3–7 days after SCI in rats, whereas this migration is further delayed in mice, only infiltrating the spinal cord after 7–14 days and doubling 2–6 weeks following SCI [40,41]. A biphasic pattern is seen both in rats and mice regarding T-cell influx [41,42]. In humans, CD8+ lymphocytes can only be found 5–10 days following SCI scattered in the spinal cord parenchyma and proliferate during the following weeks [15,43]. T lymphocytes can be divided into T cytotoxic cells (CD8+), functioning as cell-death inducers, and T-helper (Th) cells (CD4+), mainly interacting with other cells and providing molecular cues. The latter includes a variety of populations (Th1, Th2, Th17, Treg) with different targets and producing diverse cytokines and, thus, having different effects on the inflammatory environment. SCI induces a local pro-inflammatory environment that skews the T-helper lymphocytes’ balance in favour of Th1 cells [44]. These are known to produce pro-inflammatory cytokines, such as TNF-α, IL-6 and INF-γ, which lead to cell death, blood–spinal cord barrier disruption and macrophage transformation into the pro-inflammatory type (M1 phenotype) [44,45,46]. The persistence of this pro-inflammatory environment contributes to neuronal loss, despite the increased expression of neurotrophic factors in the spinal cord [45]. Although Th1 cells appear to dominate in the spinal cord tissue following SCI, Th2 cells assume a relevant role and secrete anti-inflammatory cytokines, such as IL-4, IL-10 and IL-13, which counteract the initial inflammation. In contrast to Th1 cells, Th2 cells induce M2 (anti-inflammatory) macrophage transformation (see below) [44,45,46]. Th17 cell polarization leads to neuroinflammatory changes, in a similar fashion as Th1 polarization [47]. On the other hand, Treg cells participate in tissue remodelling by suppressing the inflammatory response. In the CNS, Treg polarization may be achieved by the same molecular cues as Th1 polarization, aiding the resolution of the Th1-polarized response [48,49]. It would be interesting to explore whether pharmacological interventions that increase the concentration of Th1 and Treg cells affect SCI functional and histological outcomes.

Type B lymphocytes are also important in SCI. When released from the bloodstream, they are known to differentiate into plasma cells in several tissues, including the CNS. Plasma cells were found in the spinal cord of mice with SCI, where they seem to produce deleterious antibodies that compromise functional recovery [50]. In peripheral blood, a study showed a significant reduction in CD19+ B lymphocytes, with an apparent nadir in the first 24 h after SCI and a gradual recovery achieved by the end of the first week post-injury [24]. This leads to transient immunoglobulin deficiency [51], likely contributing to SCI-induced peripheral immune dysfunction [52].

Unlike the rest of the lymphocyte subsets, NK cells are effector cells of innate immunity. Studies showed that the NK lymphocyte blood count is decreased in chronic SCI patients, as is the expression of specific NK genes [25,26,53]. However, a recent systematic review did not confirm these observations [22].

Importantly, changes in the population of lymphocytes have been linked to SCI-induced immune deficiency syndrome (SCI-IDS), where early lymphopenia is present in SCI patients. This condition mainly depends on the reduction in B and NK lymphocytes [51] (Table 1). The lymphocyte-dependent immunosuppression observed in acute SCI patients is thought to be linked to a post-aggression syndrome, rather than having a neurologic origin [51]. This immune disruption is one of the main culprits for the frequent infections following SCI and, therefore, deeply impacts morbidity and mortality in paraplegic and tetraplegic patients [51,54]. Interestingly, the number of peripheral lymphocytes and neutrophils is strongly related to the SCI patients’ prognosis, with a higher circulating neutrophil-to-lymphocyte ratio being associated with a worse outcome following acute cervical traumatic SCI [55].

#### 3.2.3. Resident and Peripheral Macrophagic Cells: Microglia, Monocytes and Macrophages

Microglia and peripherally derived macrophages are phagocytic cells that intervene in spinal cord tissue changes following SCI [56,57]. Although they share immune markers and mechanisms, these cells have very different embryonic origins. While microglia cells originate from the yolk sac as primitive macrophages [58,59,60,61], monocyte-derived macrophages (MDM) originate from hematopoietic processes in the adult, with a mesodermal origin [62]. Microglia activation occurs moments after SCI [57]. An ameboid morphology accompanies the development of pro-inflammatory features, including the secretion of pro-inflammatory molecular mediators, like TNF-α and IL-1β [5,57,63,64]. Similarly to peripherally derived macrophages, microglia phenotypes can be described in a spectrum from inflammatory (M1) to alternative polarized (M2) [57,65].

Together with activated astrocytes, activated microglia recruits peripheral immune cells that infiltrate the spinal cord lesion site. Among these infiltrating cellular populations, monocytes differentiate into macrophages once they reach the nervous tissue [66]. Two waves of accumulating macrophages/microglia were reported in rodents. The first one peaks around 7 days after SCI and the second one, more prominent, around 60 days post-injury [67]. A study involving human specimens supports this pattern in acutely injured patients, but more studies are needed to confirm if this biphasic pattern is present in humans [15].

When, in the spinal cord tissue, blood-derived macrophages and resident microglia become morphologically indistinct, they are usually studied as one single cellular population [68]. Macrophages not only have pro-inflammatory effects but also participate in neuroprotective mechanisms [68]. A continuum between the pro-inflammatory M1 and the pro-reparative M2 phenotypes has been thoroughly described, both in CNS and other systems [66,69,70,71]. The regulation of this M1-M2 polarization is highly dependent on T helper cells. Pro-inflammatory (M1) and wound healing (M2a) macrophages predominate during the first couple of days after SCI in rodents. M1-polarized macrophages are highly active phagocytic cells that promote local sterilization and apoptotic cell removal. Afterwards, immunoregulatory (M2b) and immunosuppressive (M2c) macrophages start balancing the inflammatory environment and stabilizing the spinal cord tissue architecture [68]. Although phagocytosis is crucial for debris elimination in the setting of SCI, the persistently skewed M1 profile, seen in injured murine models, may contribute to the insufficient neuronal repair promoted by pro-reparative phenotypic macrophages [46,65,72]. The latter likely produce neurotrophic factors (like brain-derived neurotrophic factor and nerve growth factor), which could promote axonal regrowth [73,74,75]. The modulation of macrophagic polarization is one of the most promising pharmacological interventions in the context of SCI [76,77,78].

Curiously, MDMs arrive at the injury site by two different routes. One monocytic population matures into M2-polarized cells in the choroid plexuses and enters the CSF via the cerebral ventricles. These anti-inflammatory cells migrate along the ventricular system and reach the central canal, finally reaching the damaged spinal cord area [79]. Another monocytic population differentiates into M1-polarized monocytes–macrophages, entering through the injury site-associated leptomeninges [79]. Contrary to what is observed in other tissues, monocytes entering the spinal cord parenchyma via damaged parenchymal blood vessels are rare in the SCI acute phase [79,80]. One could speculate that the peculiar choroid plexus–CSF pathway contributes to the number of monocytic/macrophagic cells in the CSF of spinal cord-injured specimens [23,34,79].

A study focusing on blood leukocyte count variations in humans showed an important reduction in the monocytic population, reaching its nadir during the first day after SCI, but returning to pre-SCI basal by the end of the first week post-injury [24]. Mononuclear blood cells (lymphocytes and monocytes) appear to follow a similar pattern regarding peripheral count variation following SCI [24,51] (Table 1).

### 3.3. Involvement of Cytokines in Spinal Cord Injury

**Figure 2:**
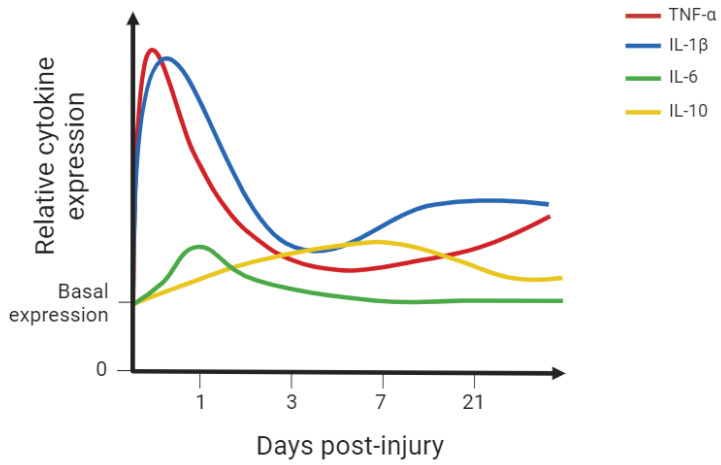
Simplified graph representing the relative cytokine expression in spinal cord tissue following spinal cord injury (SCI) in rodent models [5,63,64,81]. Some of the most studied cytokines were included. This simplified representation aims to provide the reader with a visual representation of the described findings. IL-1β: interleukin-1 beta; IL-6: interleukin-6; IL-10: interleukin-10; TNF-α: tumour necrosis factor alpha. This figure was created using BioRender.com (accessed on 8 June 2024).

The above-mentioned cellular types are recruited and exert their function by releasing a vast group of low-molecular-weight soluble peptides, proteins, and glycoproteins—the cytokines. Many of these molecular mediators are involved in inflammatory processes and several cytokines are known to play a time-dependent role in the fibrotic scar formation following SCI (Figure 2, Table 2). Cytokines also play a vital role in the vascular changes and lesion repair. Pro-inflammatory cytokines enhance inflammatory response to defend the CNS against potential microorganisms penetrating the lesion and provide a cellular environment capable of local remodelling. However, exaggerated inflammation is deleterious for neuronal growth and sprouting capacity, hence the importance of anti-inflammatory cytokines upregulation, controlling the inflammatory environment. Nevertheless, the upregulation of anti-inflammatory cytokines following SCI is modest, which hampers tissue regeneration after SCI (Figure 2). Some of the most relevant cytokines in the SCI context are explored below.

**Table 2:** Blood and cerebrospinal fluid (CSF) expression of different cytokines in human patients. The cytokines were divided into anti-inflammatory and pro-inflammatory groups. Acute spinal cord injury (SCI) was defined here by ≤14 days from lesion, and chronic SCI as >14 days from lesion.

Cytokines		Blood Expression		CSF Expression	
		Acute SCI	Chronic SCI	Acute SCI	Chronic SCI
**Pro-inflammatory**	**IL-1β**	**↓** [35,36]	**↓** [82]	**-** [36]	
	**IL-6**	**-** [36] **↑** [83,84]		**↑** [36]	
	**TNF-α**	**↑** [82]	**↑** [82]		V [85] *
**Anti-inflammatory**	**IL-4**	**↑** [35,36]		**-** [36]	
	**IL-10**	**↓** [35,36]		**-** [36]	
	**IL-13**	**-** [84]			

* This paper presents a non-statistically significant reduction in TNF expression in subacute patients (2 weeks–2 months after injury), but a non-statistically significant increase in TNF expression in late chronic patients (>24 months after injury). ↑: expression increase in comparison with controls, ↓: expression decrease in comparison with controls, -: no variation in comparison with controls, V: variable. IL-1β: interleukin-1 beta, IL-6: interleukin-6, TNF-α: tumour necrosis factor alpha, IL-4: interleukin-4, IL-10: interleukin-10, IL-13: interleukin-13.

#### 3.3.1. Tumour Necrosis Factor Alpha—(TNF-α): Inflammatory Effects

The first cytokine upregulated after SCI is tumour necrosis factor alpha (TNF-α), one of the most extensively studied cytokines. This pleiotropic cytokine, first identified in 1975, is crucial for CNS homeostasis, being expressed by multiple resident cells [21]. In physiological conditions, neurons, glial cells, microglia and astrocytes can produce TNF-α to control synaptic formation and activity and myelination, and to regulate neurotransmitter levels [86,87]. However, TNF-α is also one of the most relevant triggers for neuroinflammation, leading to blood–spinal cord barrier disruption and cell death, further potentiating inflammation by positive feedback [86].

The pro-inflammatory effects of TNF-α are evident in the setting of SCI, multiple sclerosis and other CNS conditions [5,64,88]. A recent systematic review focusing on rodent models of SCI showed that levels of TNF-α in the spinal cord increased during the first hour after SCI, paired with a slightly delayed increase in TNF receptor levels [64]. After SCI, the TNF protein is first produced by neurons, followed by astrocytes, oligodendrocytes, endothelial cells and microglia. After the first few hours after spinal injury, levels of TNF gradually reduce, although a macrophagic TNF-α production spike can be identified around the end of the first week following SCI [64].

In humans, a long-time-frame study showed that TNF-α levels in blood serum were significantly increased by SCI, after an initial reduction during the first 12 h post-injury [82]. In fact, 8 weeks after SCI, TNF-α levels had almost doubled. Another study showed a similar tendency in the CSF of SCI patients, with a slight reduction in TNF-α concentration in the early stages of disease progression, followed by upregulation of this cytokine in late chronic patients [85]. These findings seem to show that TNF-α expression in blood serum and CSF is delayed compared to its spinal cord tissue expression. One could argue that this relates to the gradual blood–spinal cord barrier dysfunction induced by TNF-α itself. Indeed, upregulation of TNF-α spinal cord tissue expression leads to blood–spinal cord barrier dysfunction and, thus, may directly promote increased TNF-α levels in blood serum and CSF.

#### 3.3.2. Interleukins IL-1β and IL-6: Inflammatory Effects

The most studied member of the interleukin-1 (IL-1) family in the context of SCI is interleukin-1 beta (IL-1β). In physiological conditions, this cytokine is produced in small amounts by microglia, astrocytes and endothelial cells. It is also expressed in the developing brain [89,90]. IL-1β takes part in several neurophysiologic processes, including regulation of neuronal survival, neurite growth and neuroplasticity [89,90,91]. Like TNF-α, IL-1β is also involved in the pathogenesis of neuroinflammatory diseases, such as multiple sclerosis and SCI [92]. In animal models of SCI, IL-1β levels increase during the first hour and peak 12 h after spinal insult [5]. In this context, this cytokine is produced by microglia, peripherally derived macrophages and astrocytes [5,63]. Interestingly, another peak in IL-1β production is identified 14–28 days after SCI, in a similar fashion as TNF-α [5,63]. Data suggests that IL-1β orchestrates an inflammatory cascade that includes molecular mediators such as interleukin-6 (IL-6) and leukemia inhibitory factor (LIF), leading to the recruitment and diapedesis of leukocytes [63].

IL-6 can also be produced in an IL-1β-independent fashion. It is constitutively produced in small amounts by resident astrocytes and microglia and positively influences neuronal growth, survival and activity in physiological conditions [93]. However, following CNS injury, IL-6 behaves as a pro-inflammatory cytokine, damaging the blood–brain barrier and promoting the differentiation of endogenous neural stem cells into the glial lineage, but not as neuronal progenitors [93,94].

A recent study compared blood and CSF cytokine levels in human patients by multiplex analysis [36] with a particular focus on IL-1β and IL-6. The study showed that levels of IL-1β in the CSF did not vary significantly in the first 14 days post-injury [36]. In contrast, the concentration of IL-1β in blood serum was significantly reduced following SCI [35,36] and plateaued between weeks 4 and 8 after injury in another study [82]. A similar trend was observed among rats [95]. The reason for this IL-1β blood serum expression pattern can only be hypothesized, but it seems that CSF and blood serum cytokine levels do not always mirror spinal cord tissue cytokine expression (Table 2). As for IL-6, its levels were elevated in CSF 3 days after SCI but reduced thenceforth. The same study demonstrated that IL-6 blood serum levels were not affected by SCI (in the first 14 days after injury), in contrast to other studies which found a transient increase 48 h post-injury [83]. The reasons for this discrepancy are not presently clear.

#### 3.3.3. Interleukin IL-10: Anti-Inflammatory Action

Interleukin-10 (IL-10) is one of the most studied anti-inflammatory cytokines, playing a critical role in healing by dampening peripheral inflammation. In the CNS, microglia and astrocytes produce IL-10 as a delayed response following an insult [96]. In fact, IL-10 production by microglia is induced by pro-inflammatory cytokines, such as TNF-α and IL-6 [97]. After SCI, IL-10 is upregulated to suppress inflammation, which occurs via the inhibition of pro-inflammatory cytokine production, deterring activation and proliferation of immune cells, and preventing pro-inflammatory polarization of macrophages and microglia [81]. IL-10 also promotes neuronal survival and affects the production of chondroitin sulphate proteoglycans, which are key elements in regulating the inhibitory properties of the glial scar [47,98]. Many studies support the idea that IL-10 spinal cord levels increase following SCI, being detected as early as 24 h post-injury and peaking 1–2 weeks post-injury, followed by a reduction, in murine models [5,81].

In humans, IL-10 levels in the CSF do not change significantly following SCI in the first 14 days post-injury [36]. In contrast, blood serum IL-10 levels were significantly reduced after SCI [35,36].

#### 3.3.4. Interleukins IL-4 and IL-13: Anti-Inflammatory Action

Interleukin 4 (IL-4) and interleukin 13 (IL-13) are related anti-inflammatory cytokines that promote tissue repair following SCI [99,100,101]. Both cytokines can induce a pro-repair phenotype in local microglia and macrophages, but are scarcely expressed in the injured spinal cord [99,101]. In murine models, IL-4 levels modestly increase following SCI, peaking 12 h post-injury. In mice, IL-13 levels remain unaffected for the first 28 days after SCI but are upregulated in rats [95,101,102,103]. Interestingly, recent studies documented conflicting observations, pointing to a significant reduction in spinal cord IL-4 and IL-13 in the same model [104]. Still, even if a slight elevation in IL-4 and IL-13 is considered, it will likely remain too low to induce conversion of microglia and macrophages into an anti-inflammatory phenotype [99,101].

In human SCI, no significant IL-4 concentration changes were observed in the CSF. Nonetheless, IL-4 blood serum concentration was significantly elevated during the first 14 days post-injury [35,36]. In rats, low blood serum levels of IL-13 were reported before and after SCI, with no significant variation [95]. Human CSF IL-13 levels were also not altered following SCI. More studies are necessary to better understand the expression variations in these cytokines and to predict potential implications of IL-4 and IL-13 modulation.

### 3.4. Beyond Interleukins: The Complement System

The complement system includes a set of proteins that participate both in innate and adaptive immunity, by orchestrating complex cascades that are involved in the regulation of chemoattraction and cell death. This intricate system can be activated by different kinds of stimuli, activating one of three distinct pathways: the alternative pathway (activated by structural components of non-self surfaces); the classical pathway (activated by antigen–antibody complexes); or the lectin pathway (triggered by binding pattern-recognition molecules to pathogen-associated molecular patterns) [105,106]. Although it is important in inflammatory processes, the complement system has also been regarded as crucial in physiologic phenomena such as development and regeneration [107]. Importantly, overactivation of the complement system is regulated by complement inhibitory proteins, such as CD55 and CD59 [108,109].

After SCI, complement proteins, such as C1q, C4, Factor B, C3, and membrane attack complex-C5b9 (MAC-C5b9), can be found in spinal cord tissue in rats [110,111,112]. The presence of complement proteins in axons or their vicinity after SCI raised the hypothesis that complement pathways may be involved in axonal degeneration and/or suppression of myelin debris by phagocytic cells [110]. Furthermore, it has been suggested that excessive activation of the complement system is critical in SCI pathogenesis [107]. Accordingly, animals with complement deficiencies showed more favourable functional and histological outcomes after SCI than wild-type animals, supporting the idea that complement activation in SCI is more deleterious than beneficial [113,114,115].

In human SCI, proteomic analysis of CSF samples also showed upregulation of complement proteins, further demonstrating their activation after spinal insult [116,117]. Likewise, increased levels of complement proteins have also been found in the serum of SCI patients [118]. Interestingly, activation of the complement system observed after SCI is also identified in other neurologic disorders, such as stroke and traumatic brain injury [107].

Despite the interesting and potentially game-changing role of the complement system in SCI, very few studies in this area of research were found, supporting the need to better explore the involvement of the complement system in SCI pathology to better understand its potential clinical value.

### 3.5. How Different Immune Systems Reflect on Different Repairing Outcomes After SCI

In humans and other mammals, SCI results in major tissue reorganization, with a lack of regeneration of lost tracts and more or less severe loss of sensorimotor function. Yet, some animals are naturally able to regenerate the injured spinal tissue. From several animal studies, it has been shown that the existence of a glial scar/bridge is vital for some degree of axonal regeneration and re-establishment of damaged neural tracts. The cellular and molecular milieu of this glial bridge seems to encapsulate the reason behind the strikingly distinct functional outcomes in humans and animals capable of natural regeneration. In agreement with the previous sections, anti-inflammatory polarization seems to provide pro-regenerative features to the glial scar and is present in animals with spinal cord regenerative abilities (Table 3). A better understanding of these mechanisms, present in naturally regenerating animals, might contribute to improving SCI treatment.

**Figure 3:**
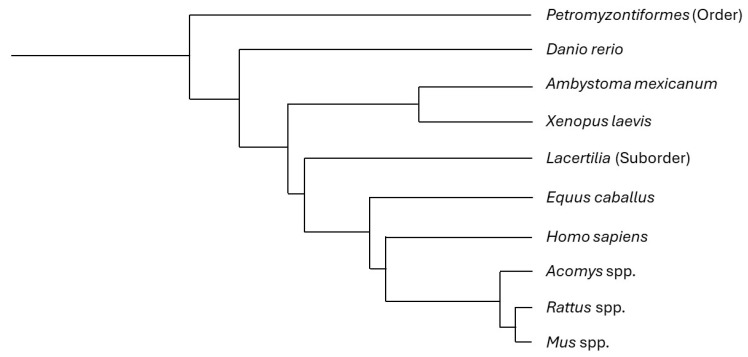
Phylogenetic tree including the most focused-on animals in this review. Some of the described animals correspond to a specific species, while others comprise different taxonomic ranks (e.g., the common name “lizard” includes all the species in the suborder *Lacertilia*, and several lizard species share the regenerative features described in this article, with no need for a discriminative approach). This tree is based on phyloT database version 2023.2 and NCBI taxonomy [119].

The following is a short description of relevant findings regarding SCI in some naturally regenerating vertebrate animals (Figure 3; Table 3), especially focusing on local tissue immune changes.

**Table 3:** Summary of the main functional and tissue differences between animals, focusing on immunologic findings. Functional and histological recovery is categorized from insignificant to total, according to the referenced works. PrM: Pre-metamorphic stages; PoM: Post-metamorphic stages.

Animal	Functional Recovery Following SCI	Histological Recovery Following SCI	Relevant Immunologic Findings (in SCI Setting)	Other Relevant Findings (in SCI Setting)
**Lamprey** [120,121,122,123]	Extensive	Some (no myelination) *	- Macrophages/microglia as neurotrophic factor producers	
**Zebrafish** [124,125,126,127]	Extensive	Some (with myelination)	- Early resolution of spinal cord inflammation	- Sox-2-positive ependymal cells
**Axolotl** [128,129,130,131,132]	Total	Total	- Early resolution of spinal cord inflammation - Poor adaptive immune response	- Sox-2-positive ependymal cells
**African clawed frog PrM** [133,134]	Extensive	Some (with myelination)	- Early resolution of spinal cord inflammation	- Sox-2-positive ependymal cells - No collagen deposition
**African clawed frog PoM** [133,134]	Insignificant	Insignificant	- Late resolution of spinal cord inflammation	- No Sox-2-positive ependymal cells
**Lizard** [135,136,137,138,139]	Extensive	Poor (without myelination)	- Increased CD59 production	- Sox-2-positive ependymal cells
**Spiny mouse** [140,141,142]	Extensive	Some (with myelination)	- Reduced pro-inflammatory profile	- Increased expression of growth factors and neural stem cell-associated genes
**Humans and most mammals** [5,15]	Insignificant	Insignificant	- Pro-inflammatory skewed profile	

* Lamprey’s nervous system lacks myelin.

#### 3.5.1. Lamprey (Order *Petromyzontiformes*)

Lampreys constitute a group of jawless fish dating back to the Devonian period, placing them among some of the oldest vertebrates that populated our planet [143,144]. These cyclostomes, which lack myelin, form an ependymal bridge uniting the stumps of the severed spinal cord, like amphibians and some teleosts [120,121,145]. The subsequent spinal cord repair leads to a robust functional recovery [122]. Studies focusing on the role of the immune system in spinal cord regeneration in lampreys are scarce, but accumulation of macrophages/microglia is observed following spinal cord injury [123]. These phagocytic cells were identified as potential producers of neurotrophic factors [73,74,75,123]. Contrary to what is seen in mammals, there are no parenchymal blood vessels in the lamprey spinal cord. Therefore, the occurrence of infiltration of peripheral immune cells, if present, cannot rely on direct spinal cord infiltration from intrinsic spinal vasculature [123,146].

#### 3.5.2. Zebrafish (*Danio rerio*)

Some teleost fish species, like the zebrafish, can partially regenerate their spinal cord after SCI, in an age-dependent fashion, with younger specimens presenting greater regenerative capacity [147,148,149]. As an animal model of SCI, the zebrafish has several advantages, including a shorter lifespan and lower maintenance costs, in comparison with mammals [124,150]. For these reasons and its remarkable regeneration ability, the zebrafish became an important animal model in regenerative medicine [124,151]. Several studies have demonstrated that after SCI, unlike humans and other mammals, the zebrafish does not produce an inhibitory glial scar, generating instead a growth-permissive bridge that allows for functional recovery [124]. Moreover, and also unlike mammals, zebrafish have GFAP+ ependymo-radial glial cells, which can differentiate into neurons in a Sox-dependent manner to replace lost or damaged ones [125,152].

Interestingly, in zebrafish, as in humans and other mammals, SCI is also followed by an inflammatory response. Neutrophils comprise the first immune cellular population arriving at the lesion site and peak 2–12 h after injury. A few hours later, macrophages and microglia accumulate in the injury site and peak 1–2 days after SCI [126,127], which promotes clearance of cellular debris. Curiously, inhibiting phagocytosis and, consequently, promoting debris accumulation did not affect axonal regeneration [126]. A study showed that pro-inflammatory cytokines, such as TNF-α and IL-1β, were upregulated in the lesion site during initial regeneration, peaking 4 h post-injury and reducing their levels afterwards. Likewise, anti-inflammatory cytokines, like TGF-β1a and TGF-β3, showed an opposing pattern, with low levels during initial regeneration and high levels later on [126].

#### 3.5.3. Axolotl (*Ambystoma mexicanum*)

The axolotl is a peculiar aquatic salamander endemic to North America [153,154]. It is considered the oldest self-sustaining laboratory animal and has caught the attention of the scientific community due to its extraordinary regeneration ability [153,154,155]. Axolotls are remarkable anamniotes that can fully regenerate their spinal cord following SCI, with no significant morphological or functional differences between already-existent and newly generated spinal tissue [128,156,157]. In these animals, spinal cord regeneration largely depends on the proliferation of ependymoglial cells, which seem critical to the organization of growing axons [130,158,159]. In axolotls, a great number of these cells resemble embryonic radial glia, extending their processes to the outer surface of the spinal cord [129,130,160,161]. In mammals, this type of cell is present as tanycytes, found in the brain ventricular system, but rarely in the spinal cord [129,130,161,162]. In axolotls and other urodeles, following SCI, the ependymoglial cells form a bulb, migrate towards the lesion centre, and promote reconnection of the cranial and caudal stumps of the injured cord [130,163]. As in lampreys, ependymal cell proliferation depends on Sox2 expression, a key factor in pluripotency [131,164]. These Sox2+ ependymal cells are known to behave as neural stem cells, repopulating the damaged spinal cord [131,164]. Unlike mammals, there is no generation of glial scar tissue in axolotls after SCI, and, thus, there is no blockade to axonal regeneration [163].

As for the immune response of axolotls to SCI, data shows that during the first 3 days post-SCI, there is upregulation of proteins involved in pro-inflammatory responses [165]. During these initial post-injury days, it is also possible to observe some leukocyte proliferation near the injury site, although axolotls exhibit a poor adaptive immune response, and the density of immune cells is not prominent [165,166,167]. Although this non-cytotoxic inflammatory response is critical to allow for tissue regeneration, this early mitigation of immune activity, paired with a poor adaptive immune response, seems important for successful scarless tissue restoration [132,167,168,169,170,171]. Nonetheless, more studies focusing on inflammatory changes after SCI are needed to better understand the mechanisms responsible for the absence of a neuron-repulsive fibrotic scar and heightened regenerative response in axolotls.

#### 3.5.4. African Clawed Frog (*Xenopus laevis*)

The African clawed frog (*Xenopus laevis)* has also been studied in the context of SCI. This amphibian, when in pre-metamorphic stages, constitutes a regenerative model, and presents Sox2-positive radial ependymoglial cells, able to differentiate in neurons after SCI [133]. However, after metamorphosis, ependymoglial Sox2 expression is lost, together with its regenerative potential [134]. During the regenerative stages in *X. laevis*, shortly after SCI, macrophages are known to infiltrate and accumulate at the injury site. This macrophage infiltration takes place earlier and resolves more swiftly during the regenerative stages in comparison with after metamorphosis. Furthermore, in non-regenerative stages, an important deposition of collagen fibres occurs at the lesion site, resembling the human glial scar [133], which does not occur in pre-metamorphic stages.

#### 3.5.5. Lizard (Suborder *Lacertilia*)

The term “lizard” is commonly used to include all squamate reptiles other than snakes and amphisbaenians, that are grouped in the *Lacertilia* suborder [172,173]. Lizards are ectothermic amniotes with exceptional regenerative capacities, as evidenced by tail repair following caudal autotomy, a feature observed in some species [174]. Caudal autotomy consists of the voluntary self-detachment of the tail and is frequently used as a defence mechanism against predators [135,174]. Curiously, microsaurs and mesosaurs are extinct animals that have been hypothesized to be able to autotomize their tails [175,176]. Since they populated the earth before dinosaurs, during the early Permian [175,176], we may consider that autotomy and, possibly, tail and spinal cord regeneration, are biological features that developed early in the phylogenetic tree but were later lost in many species.

After tail loss, tissue regeneration in lizards depends on the formation of a blastema that gradually projects from the wound. The spinal cord portion present in the tail partially regenerates, but its histology never recovers. In fact, the newly formed spinal cord lacks white and grey matter differentiation and consists of an ependymal tube surrounded by descending fibres ensheathed by meningeal components [135]. Sox-2-positive ependymal cells seem to be one of the main contributors to spinal cord regeneration, either after autotomy or after experimental spinal cord transection [135,136,137].

There are some differences regarding molecular and cellular changes between human and lizard responses to SCI. The glial scar observed in lizards does not completely block neuronal growth, contrary to what is observed in humans [137]. On the other hand, in lizards, the spinal cord inflammatory cell influx does not greatly differ from what is observed in humans: granulocytes permeate the CNS during the first days and are followed by monocytes and macrophages, which become dominant during the second week post-injury [137]. The complement system-related molecule CD59 was described as an important factor in the proximodistal axis organization of blastema formation after tail amputation in *Gekko japonicus* adults [138,139]. Similarly to what happens in mammals, in geckos, CD59 hinders cell death resulting from the activation of complement-mediated processes. After SCI, CD59 becomes particularly important in preventing neuronal death [139], and it has been hypothesized that the difference between the amount of CNS-produced CD59 by mammals and naturally regenerating lizards may contribute to the greater spinal cord regeneration capacity in the latter [139]. Considering this, it would be interesting to explore whether CD59-based treatments in mammals are effective following SCI.

#### 3.5.6. Spiny Mouse (*Acomys* spp.)

The spiny mouse is an exception among mammals, due to its reduced scarring response [141]. After SCI, the spiny mouse recovers motor and autonomic functions [142], as it generates a less repulsive environment at the injury site, contrasting with the inhibitory fibrotic scar found in other mammals [141,142]. Macrophages seem to play an essential role in the regenerative processes that take place in the spiny mouse [177]. Recent findings point to a reduced pro-inflammatory M1 profile and an increase in the expression of growth factors and neural stem cell-associated genes after SCI [140]. The suppressed but not absent inflammatory response, paired with a lack of fibrosis and stimulation of neural re-growth, enhances spinal cord regeneration, making the spiny mouse one of the most promising mammalian models to identify mechanisms that can potentiate spinal cord repair. More studies should be conducted to better understand the potential of the spiny mouse as a game-changing model in regenerative medicine.

### 3.6. Immune System-Based Therapies in the Pipeline

As explained above, several experimental and clinical studies demonstrate that an excessively pro-inflammatory local environment after SCI is deleterious for spinal cord repair and functional recovery. This has led to the development and assessment of anti-inflammatory therapies to regulate the spinal molecular environment after SCI. Some of these treatments have made their way to clinical trials, while others are still being explored in a pre-clinical setting.

#### 3.6.1. Non-Specific Anti-Inflammatory Drugs

The first immunomodulatory drug to successfully complete a phase III clinical trial was methylprednisolone [178]. Subsequent robust studies failed, however, to demonstrate functional improvement after this treatment [179,180,181]. Moreover, methylprednisolone was associated with a high rate of complications, making it a poor option for SCI treatment [179,180,181]. Interestingly, a recent clinical trial suggests that methylprednisolone and minocycline combination therapy is more effective in improving neurologic function than monotherapy [182]. Minocycline has also been tested as an individual treatment, and a phase III clinical trial focusing on minocycline, an antibiotic with immunosuppressing effects, was initiated in 2013 (NCT01828203). Although these immunosuppressing agents reduce inflammation, this occurs in a non-specific manner by targeting multiple inflammatory molecules and cells [183,184]. Indeed, methylprednisolone, a synthetic glucocorticoid, interferes with inflammatory transcriptional regulators, like NFκB and AP-1, and modulates immune cell activity and differentiation. Moreover, the anti-inflammatory effects of minocycline involve inhibition of matrix metalloproteinases, immune cell activation, and modulation of cytokine secretion. This may occur via the targeting the retinoic acid receptors [185].

#### 3.6.2. Therapies Regulating Macrophagic Polarization

Extracellular fibrotic material and deposition of some types of chondroitin sulphate proteoglycans (CSPGs) contribute to the formation of a neuronal-blocking scar, which is highly repulsive for axonal growth and regeneration of lost synaptic contacts. Digestion of this scar with chondroitinase ABC (chABC) not only promotes functional recovery [186] and axonal growth through the lesion site [187], but also favours M2-macrophage transformation, leading to an anti-inflammatory skewed profile [76] instead of an upregulation of the pro-inflammatory population of M1 macrophages. This M2-macrophage transformation is associated with enhanced production of IL-10, an anti-inflammatory cytokine [188]. A phase II clinical trial studied the effects of local injection of primed autologous macrophages on the neurologic function of acute SCI patients [189]. These macrophages were co-cultured with skin cells, a method known to induce reparative phenotypes in macrophages [189,190]. However, the analysis failed to show a statistically significant improvement in neurological function [189]. Other less-studied therapies also affect macrophage polarization, such as azithromycin, a widely used antibiotic with anti-inflammatory properties, which promoted M2 macrophage activation and improved functional recovery and tissue repair in a rodent SCI model [78]. Higenamine, a plant-derived substance used in traditional Chinese medicine, also seems to improve recovery following SCI by promoting M2-macrophage activation [77,191]. Macrophage-phenotypic modulation is also thought to be achieved by hyperbaric oxygen therapy, with many studies using SCI murine models supporting the idea that this therapy promotes a local anti-inflammatory environment that enhances spinal cord repair [192]. Hyperbaric oxygen therapy has already been explored in SCI human patients, with a few studies presenting promising results [193].

#### 3.6.3. Therapies Targeting Cytokines

Many cytokines have been associated with SCI-related inflammation, such as TNF-α. Recently, monoclonal antibodies targeting specific pro-inflammatory molecules have attracted the attention of the scientific community. Among these, an anti-TNF-α monoclonal antibody is being studied in a one-phase-I/II clinical trial (NCT04988425). Currently, anti-TNF antibodies are already used by clinicians in several inflammatory disorders, including Chron’s disease, ulcerative colitis, rheumatoid arthritis, and ankylosing spondylitis [194]. The modulation of other cytokines after SCI is also of interest, for example, by using IL-1 and IL-6 receptor antagonists, which have shown promising results in animal SCI models [195,196,197]. Similar promising results were found by administering IL-4, an anti-inflammatory cytokine, to SCI rodents, in which histologic and functional parameters were improved [99,198]. Direct cytokine modulation may represent a potentially viable and effective solution to be more thoroughly explored in the future.

#### 3.6.4. Therapies Targeting the Complement System

Modulation of the complement system constitutes a possible target to reduce inflammation following SCI. Although no human studies are available, the administration of several complement cascade inhibitors (e.g., vaccinia virus complement control protein, recombinant soluble complement receptor type 1, C1 esterase inhibitor) improved neurologic function in rodent SCI models [199,200,201]. Surprisingly, very few studies expanded these observations, and more research is needed to propel clinical trials using anti-complement therapies.

## 4. Conclusions

Spinal cord injury is associated with great suffering and is a highly demanding clinical entity, both socially and financially. Thus, it is urgent to find novel therapies that are more effective and clinically viable. Much work has been carried out to clarify SCI physiopathology, and the importance of the neuron-repulsive glial scar as a barrier to tissue repair is now well established. Its development largely depends on the inflammatory milieu generated in the injury site. Modulating the expression of specific inflammatory molecules and regulating the arrival of immune cells to the injury site presents a possible and promising way of preventing lifelong dysfunction in SCI patients. Models of animals capable of natural regeneration seem to be important for finding new molecular targets, but many potential therapies have already been suggested. It is crucial to explore advancements in experimental research and initiate clinical trials involving the most promising molecular targets.

## Figures and Tables

**Table 1:** Blood and cerebrospinal fluid (CSF) count of leukocyte subtypes in human patients. Acute spinal cord injury (SCI) was defined here by ≤14 days from lesion, and chronic SCI as >14 days from lesion.

Leukocyte Subtype	Blood Count		CSF Count	
	Acute SCI	Chronic SCI	Acute SCI	Chronic SCI
**Neutrophils**	**↑** [22] *		**↑↑** [23] †	**↑** [23] †
**T lymphocytes**	**↓↓** [24]	**-** [24,25]		
**B lymphocytes**	**↓↓** [24]	**-** [24]		
**NK lymphocytes**		**↓** [25,26]		
**Monocytes**	**↓** [24]	**-** [24]		

* Patients were not differentially analyzed according to duration of injury. † Leukocyte subtypes were not analyzed in a time-dependent fashion—the count variation presented here refers to all leukocytes (without subtype discrimination) ↑: count increase in comparison with controls, ↑↑: very expressive count increase in comparison with controls, ↓: count decrease in comparison with controls, ↓↓: very expressive count decrease in comparison with controls, -: no variation in comparison with controls. NK: natural killer. Note that the CSF of non-injured individuals does not, usually, present blood cells.

## Data Availability

The data presented in this study are available on request from the corresponding author.
